# Diseases of the Stomach

**Published:** 1892-12

**Authors:** 


### Book Reviews.

THE DISEASES OF THE STOMACH.—By C. A. Ewald, M. D., Extraordinary Professor of Medicine at the University of Berlin; Director of the Augusta Hospital, etc. Authorized translation from the second German edition, with special additions by the author, by Morris Manges, A. M., M. D., Attending Physician to Outdoor Department Mount Sinai Hospital, New York City, etc. D. Appleton & Co., New York, 1892.

THIS work which Dr. Manges has translated from the German will prove a valuable addition to the literature now in circulation on this most important subject. It is in the shape of lectures and is, in fact, based upon the stenographic reports of remarks of Dr. Ewald at the *Feriencurse fur praktische Aerzte.* Of recent years a great deal of enthusiasm has been manifested by different investigators in this particular branch of medicine, and a work containing the accumulated results of these different men would seem altogether opportune. It will be found that in the author's references he has been very liberal towards the American profession. He states that Kinnicutt, in a paper read before the Association of American Physicians, claims to have found hydrochloric acid "almost without exception," in the stomachs of fasting suckling infants, while he himself, however, believes that normally the stomach contains only traces of hydrochloric acid and cites a noted German authority as agreeing perfectly with him in his results. In his chapter on electrization of the stomach, he refers to experiments made by Pepper and reported in an article in the *Philadelphia Medical Times*, in 1871. He also says, "it is well known that spastic strictures may appear throughout the whole length of the œsophagus, and at times may become so marked as to simulate the symptoms of hydrophobia," and cites such a case reported by Barnes, an American physician, in 1882. In the chapter on Differential Diagnosis of Cancer, he refers to the report of a case by Stoner in *Boston Medical and Surgical Journal*, in 1872, in which almost the entire stomach underwent colloid degeneration without causing any marked disturbances of digestion and vomiting. In giving the etiology of gastric ulcer, he mentions the celebrated case reported by an American physician, in a paper published in Boston, 1833, entitled "Experiments and Observations on the Gastric Juice and the Physiology of Digestion," and says, "in proof of this—the transient hemorrhages and follicular suppuration due to irritating ingesta—we possess a classical witness for all time in the Canadian experimented on by Beaumont." He further speaks of a case in connection with hematemesis reported by Welch in Johns Hopkins Hospital Bulletin No. 1, of a man 50 years of age, in whom he found a ruptured miliary aneurism on a branch of the gastric artery, which gave rise to a rapidly fatal and very profuse gastric hemorrhage; and lastly, the effect of diseases of other organs upon the stomach and their reciprocal action as manifested in structural changes, as for instance, the relation between advanced atrophy of the gastric mucosa and pernicious anæmia, is mentioned by him, but adds that Henry and Osler have already called attention to this fact. It is but just to say that the translator has given to the American profession a very valuable work and one which will doubtless find a ready sale.

